# A Precious Puzzle: Unveiling a Suspected VACTERL (Vertebral, Anal, Cardiovascular Malformations, Tracheo-esophageal Fistula, Renal Anomalies, and Limb Defects) Association in a 28-Day-Old Neonate

**DOI:** 10.7759/cureus.70143

**Published:** 2024-09-25

**Authors:** Asfia Shabnam, Arumugam Vasugi, Leena Dennis Joseph, Raja Rajan, Mitila T

**Affiliations:** 1 Pathology, Sri Ramachandra Institute of Higher Education and Research, Chennai, IND; 2 Pediatrics, Sri Ramachandra Institute of Higher Education and Research, Chennai, IND

**Keywords:** anomaly, atresia, malformations, vacterl, very preterm

## Abstract

VACTERL is an acronym that stands for a wide range of congenital anomalies, including vertebral anomalies, tracheoesophageal fistula, anorectal anomalies (anal atresia), cardiac anomalies, renal anomalies, and limb malformations. While not a true syndrome, the co-occurrence of these findings suggests a common underlying cause. Here, we present a case of a 28-day-old neonate with suspected VACTERL association. The baby was delivered preterm to non-consanguineous parents. The baby had vertebral abnormalities, renal defects, an imperforate anus, a posterior urethral valve, and an absent right pulmonary artery. The current case report emphasizes the critical role of pathologists who specialize in the dissection and confirmation of fetal anomalies as members of the multidisciplinary team that manages complex pregnancies with this type of abnormality.

## Introduction

VACTERL is an acronym denoting a disorder characterized by the spontaneous, non-random correlation of particular birth abnormalities affecting numerous organ systems: vertebral, anal, cardio-vascular malformations, tracheo-esophageal fistula, renal anomalies, and limb defects [1]. VACTERL association, first described in the early 1970s, is characterized by the occurrence of at least three of the following congenital malformations: vertebral defects, anal atresia, cardiac defects, tracheo-esophageal fistula, renal anomalies, and limb deformities [2]. In addition to these primary characteristics, children may have various congenital anomalies such as hemifacial microsomia, external ear malformations, lung lobation problems, intestinal malrotation, and genital anomalies. The incidence is estimated to be between one in 10,000 and one in 40,000 live births. We present one such case which includes congenital heart abnormalities, features of acute lung injury, and spinal deformities. The etiology is uncertain; however, it is thought to be complex [3]. They can be associated with some known chromosomal abnormalities, like trisomy 13, 18, and 5p syndrome [4]. Recent research indicates that VACTERL may be caused by faulty SHH (Sonic hedgehog system) signaling during human embryogenesis [5]. The reason it is labeled as an association rather than a syndrome is that, while all of the birth problems are linked, it is unclear which genes or sets of genes cause these birth defects to manifest. A disturbance in the developing mesoderm in the first 4-5 weeks after conception (during blastogenesis) has been proposed as the basis for such a non-random connection [6].

## Case presentation

A two-hour-old very preterm first-order male child born to a non-consanguineously married couple was admitted to the NICU of our institute. He was born at a gestational age of 30 weeks + 2 days by lower segment cesarean section following premature rupture of membranes. There was no history of congenital abnormalities in the family. 

Obstetric history

**Figure 1:**
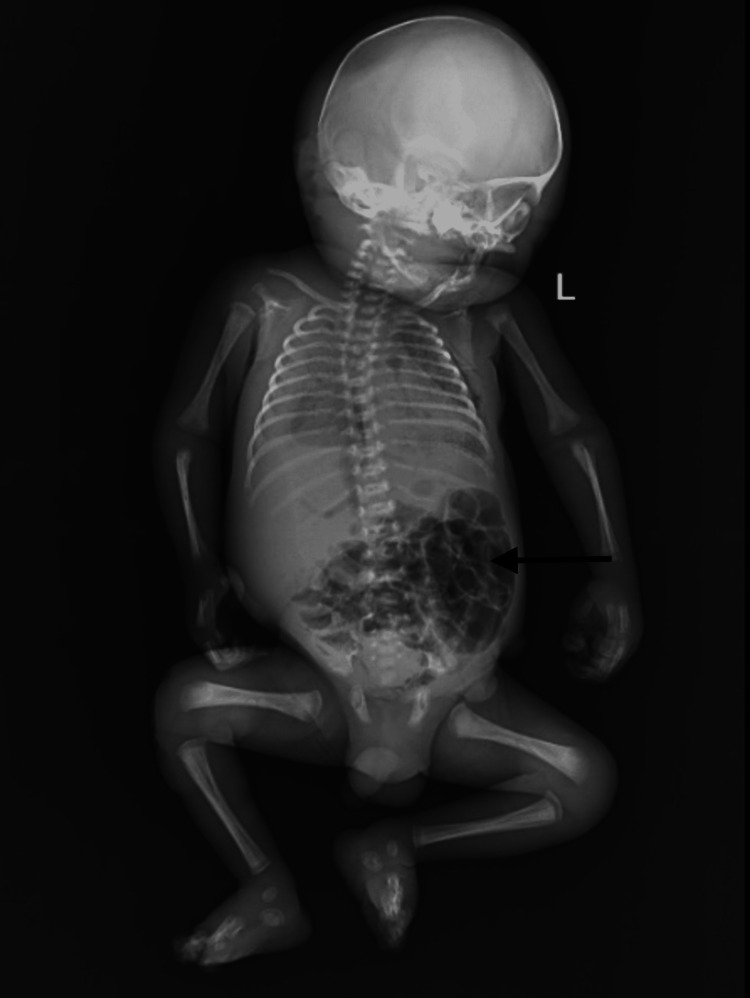
Fetal X-ray depicting absent bowel gas shadows (arrow) in the rectum consistent with an imperforate anus

**Figure 2:**
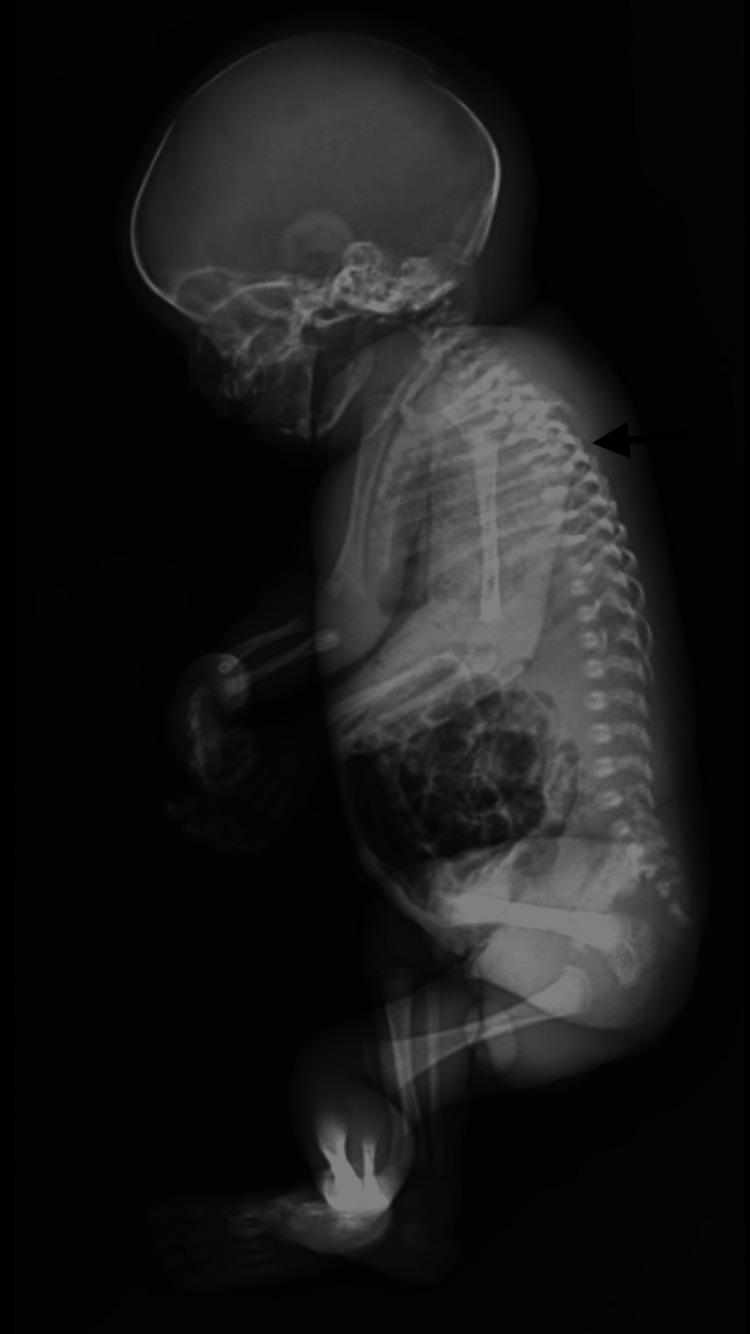
Fetal X ray showing butterfly vertebrae at the mid-thoracic level and portal venous gas shadows

**Figure 3:**
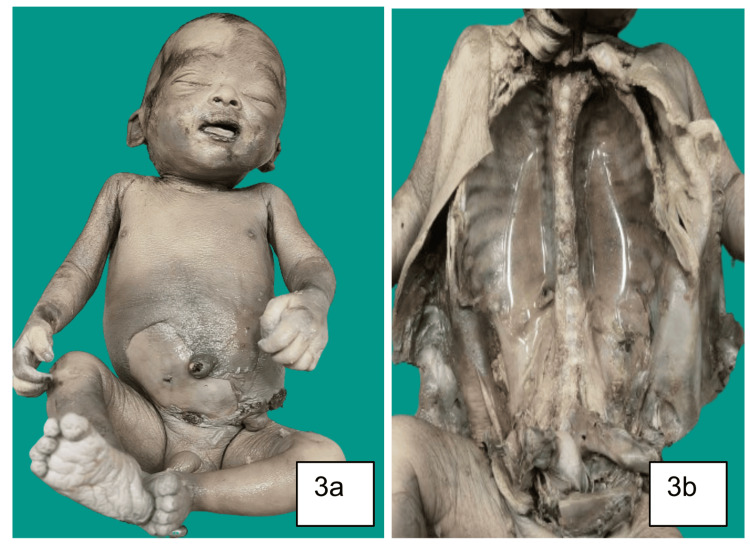
(A) The male fetus, colostomy site visible externally. (B) The vertebral column showing features of hemivertebrae. The remaining organs appeared normal grossly

**Figure 4:**
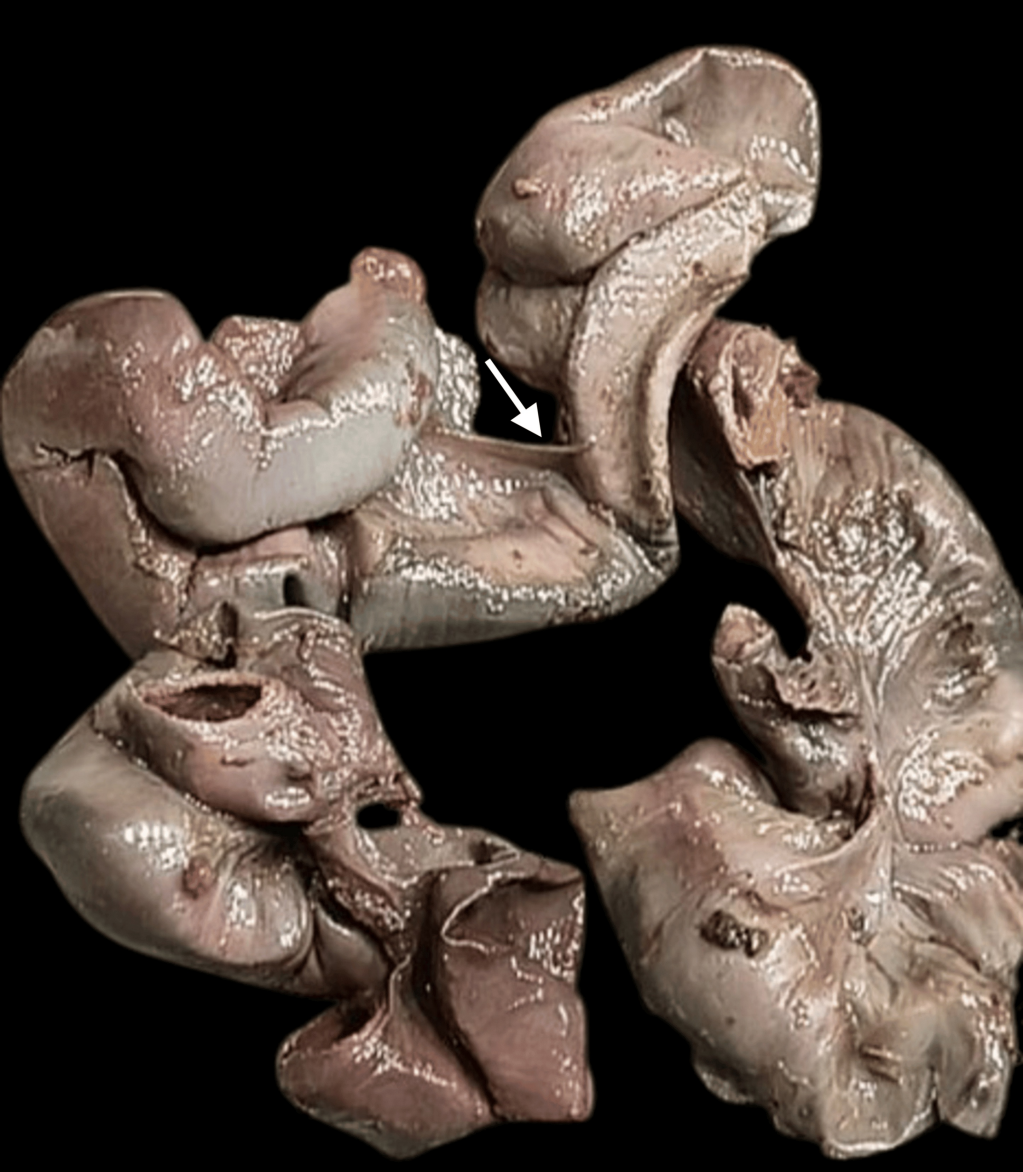
Gross examination of the intestines showing thinning of the mucosa (arrow)

A 32-year-old Primi woman presented for a routine prenatal ultrasound scan at 30 weeks gestation. The ultrasound revealed the presence of a single umbilical artery with a potential vertebral body malformation in the thoracolumbar spine and enlarged echogenic kidneys, suggestive of renal pyelectasis. The amniotic fluid index could not be assessed. Another significant finding was mild shortening of the femur bones. Amniocentesis was offered to rule out chromosomal abnormalities, but the parents declined. The pregnancy continued and the fetus was delivered very preterm at 30 weeks + 2 days via cesarean section due to breech presentation and premature rupture of membranes. On postnatal examination, the neonate displayed anal stenosis, requiring surgical correction. The echocardiogram revealed pulmonary hypertension and an absent right pulmonary artery. Further radiological investigations (Figures 1, 2) confirmed the prenatal suspicions. X-ray revealed a butterfly vertebra at the mid-thoracic level. Renal ultrasound confirmed bilateral renal dysplasia and obstructive uropathy. CT revealed an absent right pulmonary artery (Figure 3). The baby spent three weeks in the NICU and underwent corrective surgery for the imperforate anus. The baby was survived by the colostomy site. By day 3 of life, it was noted that the baby was not passing urine. During surgery, the surgeons discovered a posterior urethral valve obstructing the passage of urine and a subsequent conduit was made from the bladder for passage of urine. The baby eventually developed sepsis. He had tachypnea with labored breathing and his SpO2 was maintained with 5 liters of oxygen. He remained ventilator-dependent until complications from intestinal perforation and sepsis resulted in his death. On autopsy, external examination showed peeling of skin, facial features were normal, and the colostomy site was noted in the abdomen due to an imperforate anus (Figures 3a, 3b). Internal examination revealed extensively thinned out and grey black intestine (Figure 4). The right pulmonary artery could not be traced and the lungs appeared hard and congested.

**Figure 5:**
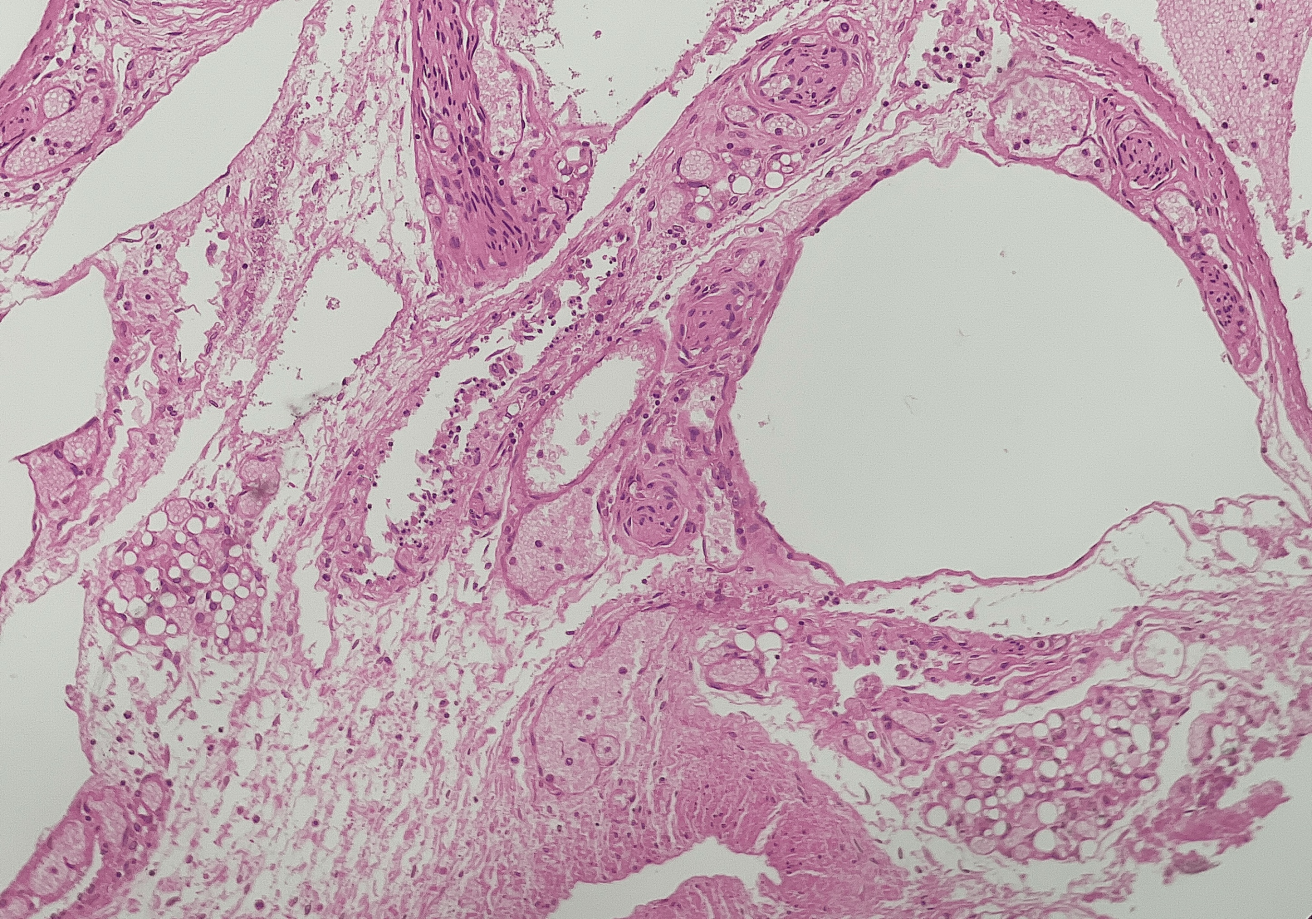
H&E-stained image of intestines showing extensive necrosis and hemorrhage with empty submucosal spaces depicting pneumatosis coli (200X)

**Figure 6:**
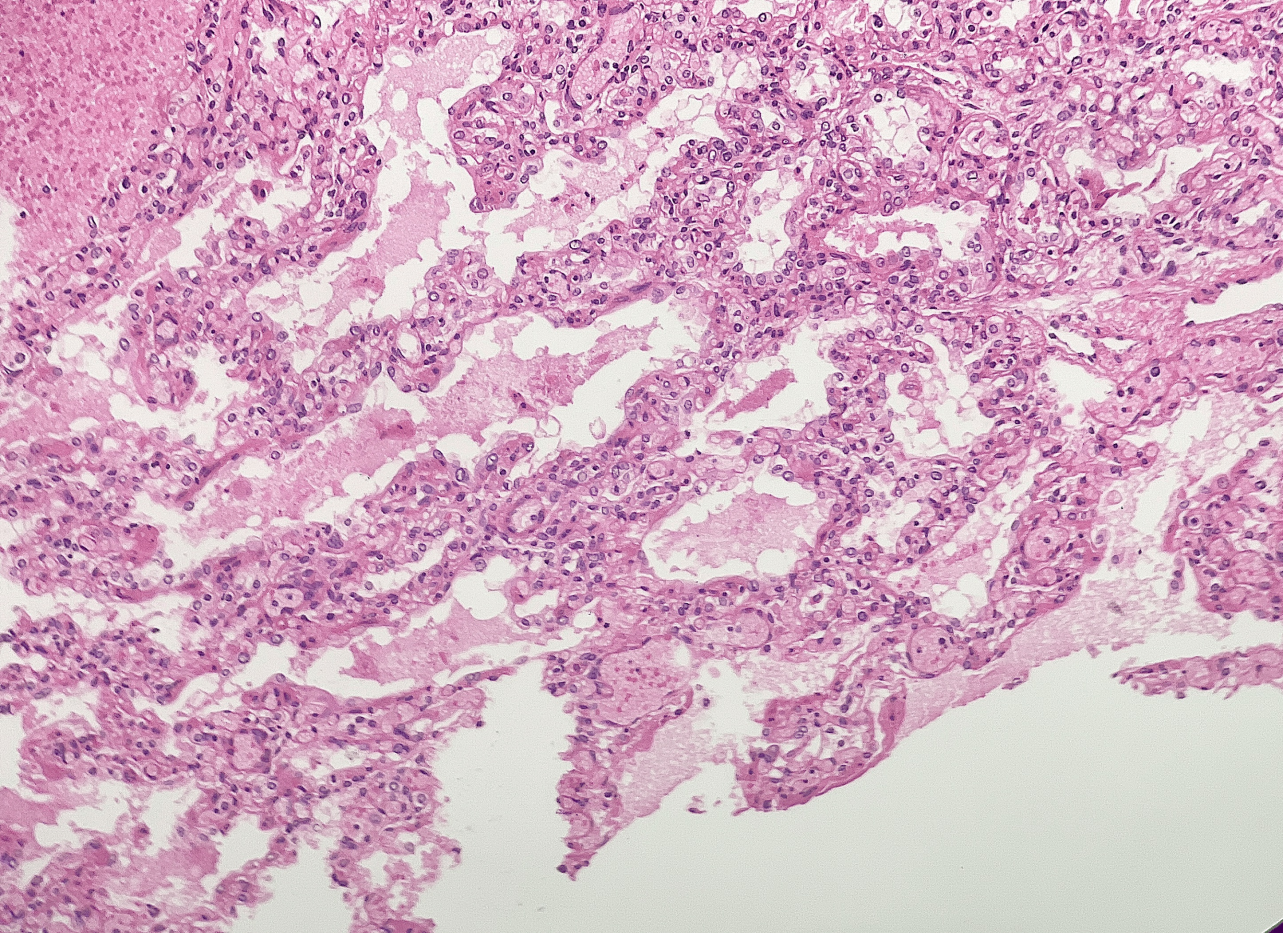
H&E-stained image of left lung parenchyma showing interstitial congestion and pneumocyte hyperplasia suggestive of acute lung injury (200x)

The liver and spleen were congested, with the spleen exhibiting areas of autolysis. The stomach demonstrated autolytic changes, and the intestines showed extensive and widespread necrosis, bleeding, extracellular mucus, inflammation with leukocytosis, and empty cyst-like spaces in the lining consistent with necrotizing enterocolitis (Figures 5, 6).

Both kidneys and adrenal glands showed signs of autolysis with some areas of cell death. The left lung had congested tissue, pneumocyte hyperplasia and a protein-rich fluid buildup suggesting acute lung injury (Figure 6).

The right lung had an abscess (collection of pus) with surrounding tissue damage consistent with acute lung injury. The reproductive organs showed signs of autolysis while the neck structures, heart, and brain appeared normal. Bone development and maturity were normal for the fetus age. 

## Discussion

VACTERL association is a complex and rare congenital condition with varied etiological factors and presentations. At least three morphological features for the diagnosis, without clinical or laboratory evidence of overlapping conditions, while few emphasize the presence of certain features, especially tracheo-esophageal fistula or anorectal malformation [2]. The etiological factors and pathogenesis are unclear, but a defect in mesodermal differentiation due to varied causes (environmental, genetic, and multi-factors) is implicated in the early first trimester [7,8]. 

Vertebral anomalies amount to 70% of patients with VACTERL association. They range from hypoplastic vertebrae to hemivertebrae. The present case was diagnosed to have butterfly vertebrae in radiology. These anomalies rarely cause difficulties in early life while they are at risk of developing scoliosis in the later stages of life [9]. Imperforate anus or anal atresia is seen in 55% of cases of VACTERL. Cardiovascular abnormalities are seen in 30% of cases of VACTERL association. The common defects are ventricular septal defect, atrial septal defect, and tetralogy of Fallot. Less common ones include transposition of great arteries and truncus arteriosus. Tracheoesophageal fistula or esophageal atresia is seen in 70% of cases. Renal defects include obstructive uropathy or malformation of one or both kidneys and are seen in 50% of cases. Limb defects include polydactyly, absent or displaced thumb, syndactyly, leg, and forearm defects including radial aplasia, and are seen in 70% of cases. Other less common features seen in VACTERL association include lung lobation defects, intestinal malrotation, external ear malformations, facial asymmetry including hemifacial microsomia, and genital anomalies [10].

Embryological studies suggest that there are multiple polytopic developmental field defects, a disruption in the developing mesoderm in the early weeks of embryogenesis. They can also be associated with some known chromosomal abnormalities, like trisomy 13, 18, and 5p syndrome [11]. Other etiological possibilities are a diabetic mother, a baby born out of infertility treatment, and exposure to lead, anticonvulsants, estrogen or progesterone-containing compounds, or alcohol during the period of embryogenesis causing mutations in the genes like HOXD13, FOXF1, and ZIC3. VACTERL association has overlapping features with other syndromes like CHARGE syndrome, Feingold syndrome, 22qll deletion syndrome, Pallister-Hall syndrome, Townes-Brocks syndrome, caudal regression syndrome, Fanconi anemia spectrum, electrodactyly-ectodermal dysplasia syndrome, Goldenhar Syndrome, Nager syndrome, sirenomelia, Jarcho-Levin syndrome and Klippel- Fiel syndrome [12].

Diagnosis depends on the phenotypic features and radiology. However, detecting prenatally through ultrasound or echocardiogram and chorionic villi sampling is very difficult as the etiological factors of the VACTERL association are unclear. Radiological features that may suggest VACTERL prenatally include dilated colon, vertebral defects, polyhydramnios, absence of gastric bubbles, and limb abnormalities [13]. Management of infants depends on a multidisciplinary approach involving orthopedic surgeons, urologists, neonatologists, cardiologists, and otorhinolarngologists. Prognosis depends on the severity of the defects and the effective surgical management. However, considerable medical challenges persist throughout life.

## Conclusions

VACTERL anomaly is a relatively uncommon congenital abnormality. Prenatal imaging frequently misses some characteristics of the VACTERL such as anorectal malformation and tracheoesophageal fistula. The prognosis and early neonatal management of the condition depend mainly on the severity of malformations ascertained prenatally. Therefore, a major contribution to the diagnosis of VACTERL connection is made by fetal autopsy. This case highlights the significance of considering VACTERL association in patients with multiple congenital anomalies, especially those affecting the vertebral column, heart, trachea/esophagus, kidneys, and limbs. Early diagnosis, ideally prenatal, followed by timely intervention for each specific anomaly can substantially enhance the patient's prognosis and quality of life. In certain instances where the mother and fetus are at risk, early identification of VACTERL association is imperative to provide the patient with the option of terminating the pregnancy. Further research is necessary to clarify the underlying causes of VACTERL association and explore potential preventive strategies.
